# The relationship between duration of subjective poverty and health among Chinese adults: Evidence from the China Family Panel Study

**DOI:** 10.3389/fpubh.2022.939569

**Published:** 2022-10-05

**Authors:** Dan Cao, Zhongliang Zhou, Yangling Ren, Qiwei Deng, Xiaohui Zhai, Guanping Liu, Dantong Zhao, Yaxin Zhao, Chi Shen

**Affiliations:** ^1^The First Affiliated Hospital of Xi'an Jiaotong University, Xi'an, China; ^2^School of Public Policy and Administration, Xi'an Jiaotong University, Xi'an, China; ^3^School of Public Administration, Southwestern University of Finance and Economics, Chengdu, China; ^4^School of Public Health, Xi'an Jiaotong University, Xi'an, China

**Keywords:** subjective poverty, health, self-rated health (SRH), depressive symptoms, China

## Abstract

**Introduction:**

The disadvantaged socioeconomic status could have accumulated negative effects on individual. In the Chinese context, studying subjective and relative poverty is more important under the implementation of the Targeted Poverty Alleviation campaign. This study aims to provide evidence of the relationship between the duration of subjective poverty and both physical and mental health among Chinese adults, using nationally longitudinal data from 2010 to 2018.

**Materials and methods:**

Data were extracted from a nationally representative survey database—the China Family Panel Study (CFPS). The total sample size contains 12,003 adults, with 3,532 in the urban area and 8,471 in the rural area. Self-rated health and depressive symptoms were set as indicators of physical health and mental health, respectively. The duration of subjective poverty was measured by self-rated income level in the local area from 2010 to 2016. A series of ordinary least square regression was adopted to measure the relationship between duration of subjective poverty and health.

**Results:**

For the urban residents, the average duration of subjective poverty is 1.99 time points, while 1.98 time points for the rural residents. Net of objective poverty, duration of subjective poverty has a significantly negative association with individual's self-rated health in the rural sample (Coef. = −0.10, *p* < 0.001). Compared with those who have not experienced subjective poverty, the self-rated health score of people who experienced four time points is likely to decrease by 0.54 in the rural area and 0.30 in the urban area. In terms of mental health, 1 unit increase in the duration of subjective poverty is related to 0.15 unit increase in Center for Epidemiologic Studies Depression Scale-8 (CES-D8) scores in the urban sample and 0.46 in the rural sample. Compared with those who have not experienced subjective poverty, the CES-D8 scores of people who experienced four time points are likely to increase by 1.47 in the rural area and 0.95 in the urban area.

**Conclusion:**

A longer duration of subjective poverty has a cumulatively negative effect on Chinese residents' physical and mental health, especially in rural area. Our study advocates researchers and policymakers pay more attention to the cumulative effect of subjective poverty on health.

## Introduction

Income level and socioeconomic status (SES) have long been a research focus, as they may be related to a series of health outcomes. Low income or lack of material resources is defined as poverty (1). One of the most important outcomes caused by socioeconomic inequality is health inequality. Plenty of literature has documented that there was a considerable gap in not only health status but also health utilization and related expenditure between the poor and the rich (2–4). It was proposed that the poverty population was found to die earlier and to be more vulnerable to ill health (5). In developing countries, health inequality caused by low income and low SES is more severe. Disadvantaged SES could have accumulate negative effects on individuals (6). People with low SES possess fewer resources such as education, health, and social capital; on the contrary, people with high SES have more access to better resources which can be accumulated through their life course.

From the life course perspective, it is important to investigate the health damage of not only the present disadvantaged SES but also the duration of socioeconomic disadvantage in the past (7, 8). On one hand, long duration of poverty could have different effects on health compared with a short duration of poverty; on the other hand, health inequity is cumulatively shaped by present and past SES (9). Research focusing on contemporary poverty analysis may neglect those who have experienced poverty for a long time, while their living materials are kept being deprived (10). People who expose to chronic poverty are strongly predicted to have poor mental health and functional impairment (11). Krysia reported that a longer duration of poverty was related to a heavy drinker and more frequent heavy drinking in later life, which were both risk factor for physical health (8).

The world has been dedicated to reducing poverty over the past decades. China has also implemented an anti-poverty program called the Targeted Poverty Alleviation campaign (TPA) since 2014. The government employed several efforts to directly target the poor households; for example, the village cadres would visit households and interview them to accurately identify the poor (12). In addition, the government also made a detailed poverty alleviation plan for each poor household, including a series of subsidies in agriculture and health utilization (12). In 2021, China announced that it had achieved the goal of the anti-poverty program and eliminated absolute poverty. In such context, studying objective and absolute poverty has less significance than subjective and relative poverty in China. Subjective social status (SSS) captures not only the disadvantaged social resources but also *psychological pain* of people which could also affect health (13). It is usually assessed by asking about the individual's sense of their standing or economic status on the social ladder (14). SSS is a more comprehensive measure of individual's social status than objective social status (OSS) (15).

It has been largely documented that SSS is a great contributor to physical and mental health (16, 17). For example, Kim and colleagues found that in Hong Kong, lower SSS-community scores were significantly associated with greater cognitive decline among older adults (18). Another research conducted in rural South Africa also found that higher subjective social position predicted higher cognitive scores (19). A study conducted in both Japan and USA indicated that SSS could negatively affect self-rated health, while neuroticism and sense of control mediated this relationship (20). In terms of mental health, a population-based study from Hong Kong suggested that the subjective poverty population had severer mental distress and thus had worse mental health; it also implied that reducing subjective poverty could be a more effective way to improve mental health than reducing objective poverty (21). The most common explanation to theoretically support the relationship between subjective poverty and health is the social psychological mechanism. Subjective poverty reflects individual subjective economic status when compared with others. Therefore, low SSS or subjective poverty may be related to loneliness and social isolation (22) and thus affect the mental health of individual, such as depressive symptoms. Additionally, the negative effect subjective poverty brings to personal emotion and psychological conditions may furtherly influence one's immune system (23).

SSS could be different at different time points, and previous studies had already documented that it could have a cumulative relationship with cognitive function decline for older adults in China (24). Despite the ample evidence of the relationship between SSS and health, there are several research gaps. First, research on SSS in China is rather limited, although it became more important with the implementation of TPA (25). Second, evidence of the duration of SSS and physical and mental health is insufficient, especially in developing countries like China, where health inequity is severer. Existing literature cares more about cross-sectional subjective poverty, instead of using longitudinal data measuring chronic subjective poverty. Therefore, this study aims to provide evidence of the relationship between the duration of subjective poverty and both physical and mental health among Chinese adults, using a nationally longitudinal data from 2010 to 2018; such evidence will help to better understand whether subjective poverty has cumulative effects in health in China's context.

## Materials and methods

### Data sources

Data used in this study was extracted from a nationally representative survey database conducted by Peking University—the China Family Panel Study (CFPS) 2010–2018. It covered 25 provinces or municipalities and autonomous regions in China (26). A multistage probability proportional to size sampling method was used in CFPS. The first-stage sampling unit was district or county; the second-stage sampling unit was community or village; and the third-stage sampling unit was household. Education experiences, marriage, work, retirement and pension, daily activities, social network, health, and behaviors were all surveyed in CFPS.

The baseline survey was conducted in 2010, and subsequent surveys were conducted in 2011, 2012, 2014, 2016, 2018, and 2020. The 2011 survey was a small-scale interview survey, and the 2020 database has not been fully released to the public. Therefore, 2010, 2012, 2014, 2016, and 2018 surveys were adopted in this current study. Data from 2010 to 2016 were used to assess the duration of subjective poverty and objective poverty. After excluding those who were lost follow-up, who were under 18, and whose subjective poverty status in any wave was missed value, our total sample size contains 12,003 adults, with 3,532 in the urban area and 8,471 in the rural area.

### Dependent variables

#### Self-rated health in 2018

We measured the self-rated health (SRH) of Chinese adults in 2018 as the indicator of present physical health. SRH is a strong and widely used predictor of illness and physical health (13). It has been validated to be related to physical functioning and mortality (27, 28), and it has also been adopted in a previous study studying the relationship between SSS and health using CFPS (29). In CFPS, respondents were asked by the question “How do you rate your overall health status?”. The answer to this question contains five categories, “poor,” “fair,” “good,” “very good,” and “excellent,” with values from 1 to 5.

#### Depressive symptoms in 2018

Depressive symptoms in 2018 are used to measure present mental health. It has been proved to be an effective indicator to measure individual's mental health (30). In CFPS 2018, the Center for Epidemiologic Studies Depression Scale-8 (CES-D8) was deployed to measure the depressive symptoms of respondents. In CES-D8, respondents are asked to answer how often in the past week they felt for eight items, including feeling depressed, feeling everything he did was an effort, sleeping restless or not, happy, feeling lonely, enjoying life, feeling sad, and unable to get going. The choices are ranging from “none or almost none of the time” to “all or almost or all of the time,” with values 0–3. Total scale scores are from 0 to 24, with higher scores representing a higher frequency of depressive symptoms. The reliability and validity have been documented in previous studies (31, 32).

### Independent variables

*Duration of subjective poverty from 2010 to 2016* We measured the duration of subjective poverty using CFPS 2010 to 2016. In each wave, the respondents were surveyed with the question: “How do you rate your income level in your local area?” The five available options are from “the lowest” to “the highest” with values from 1 to 5. We categorized individuals whose answers were “the lowest” as experiencing subjective poverty (24). With four waves from 2010 to 2016, the duration of subjective poverty is grouped as zero/never, one time point, two time points, three time points, and four time points.

### Control variables

#### Present subjective poverty

We also controlled the present subjective poverty status in 2018 wave in our regression. A binary variable measures whether the respondents rated their income level in the lowest level (income level in the lowest level: present subjective poverty = 1).

#### Duration of objective poverty from 2010 to 2016

We also measured the duration of objective poverty using CFPS 2010 to 2016, to evaluate the effect of subjective poverty independent of objective poverty. The objective poverty was defined as the respondents' annual household income per capita being below the mean value of the lowest income quintile in each survey wave, consistent with a previous study (24). With four waves from 2010 to 2016, the duration of objective poverty is grouped as zero/never, one time point, two time points, three time points, and four time points. The mean values of the lowest income quintile in each survey wave were derived from the China Statistical Yearbooks. Urban residents and rural residents were grouped separately, due to the large income gap between urban and rural areas in China. For urban residents, the average value of the lowest 20% household income per capita was 7617 CNY in 2010, 10,354 CNY in 2012, 11,219 CNY in 2014, and 13,004 CNY in 2016; for rural residents, the average value of the lowest 20% household income per capita was 1,870 CNY in 2010, 2,316 CNY in 2012, 2,768 CNY in 2014, and 3,007 CNY in 2016^1^.

#### Present objective poverty

The present objective poverty in 2018 was also included in our regression. A binary variable measures whether the respondent's household income per capita was below the mean values of the lowest income quintile in 2018 (household income per capita below the mean values of the lowest income quintile: present objective poverty = 1). For urban residents, the average value of the lowest 20% household income per capita was 14,387 CNY in 2018; for rural residents, the average value of the lowest 20% household income per capita was 3,666 CNY in 2018, also obtained from the China Statistical Yearbook^1^.

#### Other covariates

Some other covariates that may potentially affect physical and mental health were also included in our analysis (32, 33). Marital status was categorized into three groups: unmarried, married, and other statuses. Body mass index (BMI) was grouped into four groups, with 18.5–23.9 as standard for Chinese people, <18.5 as underweight, 24–27.9 as overweight, and ≥ as obese (34, 35). Variable education contained four groups according to respondent's highest education qualification: illiterate or semi-illiterate, junior school and below, high school and technical secondary school, and junior college and above. Age was categorized into five groups, including 18–35, 36–45, 46–55, 56–65, and 66 and above. Rural residents were identified using the hukou system. Other variables included currently working, region, and gender.

### Statistical analysis

We conducted a series of ordinary least square (OLS) regression models to analyze the relationship between the duration of subjective poverty and health. Due to the different living contexts in rural and urban areas, both rural and urban samples were analyzed separately. During the analyzing process, first, we controlled covariates in the regression model to see the coefficient of the duration of subjective poverty. Second, we added the present subjective poverty as a control variable to see whether the coefficient would change. Third, we furtherly added the duration of objective poverty and present objective poverty. All data analyses were conducted using Stata 15.0.

In each regression model, we calculated the variance inflation factor (VIF) to examine the collinearity. The results are shown in Supplementary Tables A1, A2 in the appendix. The VIFs are always <10, indicating that there is no collinearity in our models.

## Results

### Descriptive results

The definition and descriptive results in 2018 are shown in Table 1. The average value of SRH of the total sample is 2.80, with 2.78 in the urban area and 2.81 in the rural area. The mean CES-D8 score of the total sample is 5.47, and that of the urban sample is 4.71, and rural sample, 5.80. The average duration of subjective poverty is 1.99 time points for the urban residents, and 1.98 time points for the rural residents. About 27.1% of urban residents were experiencing subjective poverty in 2018, and 27.3% of rural residents were experiencing subjective poverty in 2018. In terms of objective poverty, the average duration of objective poverty from 2010 to 2016 is 1.05 time points for the urban residents and 0.58 time points for the rural residents, respectively. About 14.4% of urban residents were objectively impoverished in 2018, while 11.3% were rural. The average age of the total sample is 54.17 years old, with 55.55 in the urban area and 53.59 in the rural area.

**Table 1:** Characteristics in 2018.

**Variable**	**Definition**	**Total sample**		**Urban sample**		**Rural sample**	
		**Observation**	**Mean (S.D.)**	**Observation**	**Mean (S.D.)**	**Observation**	**Mean (S.D.)**
Self-rated health status	Ranges from 1 to 5 from *very bad* to *very good*	1,2002	2.80 (1.22)	3,532	2.78 (1.07)	8,470	2.81 (1.28)
Depressive symptoms	Ranges from 0 to 24	1,1914	5.47 (4.08)	3,514	4.71 (3.76)	8,400	5.80 (4.16)
Duration of subjective poverty	Ranges from 0 to 4	1,2003	1.98 (1.31)	3,532	1.99 (1.41)	8,471	1.98 (1.27)
Present subjective poverty	=0 if not*; =1 if yes	1,1848	0.27 (0.45)	3,503	0.27 (0.45)	8,345	0.27 (0.45)
Duration of objective poverty	Ranges from 0 to 4	9,753	0.73 (1.06)	3,056	1.05 (1.51)	6,697	0.58 (0.89)
Present objective poverty	=0 if not*; =1 if yes	1,2000	0.12 (0.33)	3,529	0.14 (0.35)	8,471	0.11 (0.32)
Working status	=0 if not working*; =1 if currently working	1,2003	0.77 (0.42)	3,532	0.57 (0.50)	8,471	0.85 (0.36)
Education level	=0 if illiterate or semi-illiterate*; =1 if junior school and below; =2 if high school and technical secondary school; =3 if junior college; =4 if bachelor and above	1,2003	1.01 (0.83)	3,532	1.55 (0.93)	8,471	0.78 (0.66)
Marital status	=0 if unmarried*; =1 if married; =2 if others	1,1973	0.18 (0.56)	3,526	0.22 (0.62)	8,447	0.17 (0.54)
BMI group	=0 if normal*; =1 if underweight; =2 if overweight; =3 if obese	1,2003	1.03 (1.13)	3,532	1.08 (1.13)	8,471	1.01 (1.12)
Gender	=0 if female*; =1 if male	1,2003	0.51 (0.50)	3,532	0.52 (0.50)	8,471	0.50 (0.50)
Region	=0 if western area*; =1 if middle area; =2 if eastern area	1,1994	1.22 (0.81)	3,531	1.40 (0.72)	8,463	1.15 (0.84)
Age		1,2003	54.17 (12.55)	3,532	55.55 (13.36)	8,471	53.59 (12.15)
Age group	=0 if ≥18 and ≤ 35*; =1 if ≥36 and ≤ 45; =2 if ≥46 and ≤ 55; =3 if ≥56 and ≤ 65; =4 if ≥66	1,2003	3.41 (1.19)	3,532	3.52 (1.22)	8,471	3.37 (1.18)
Rural	=0 if in urban area*=1 if in rural area	1,2003	0.71 (0.46)				

*Reference group for all regressions.

**Figure 1:**
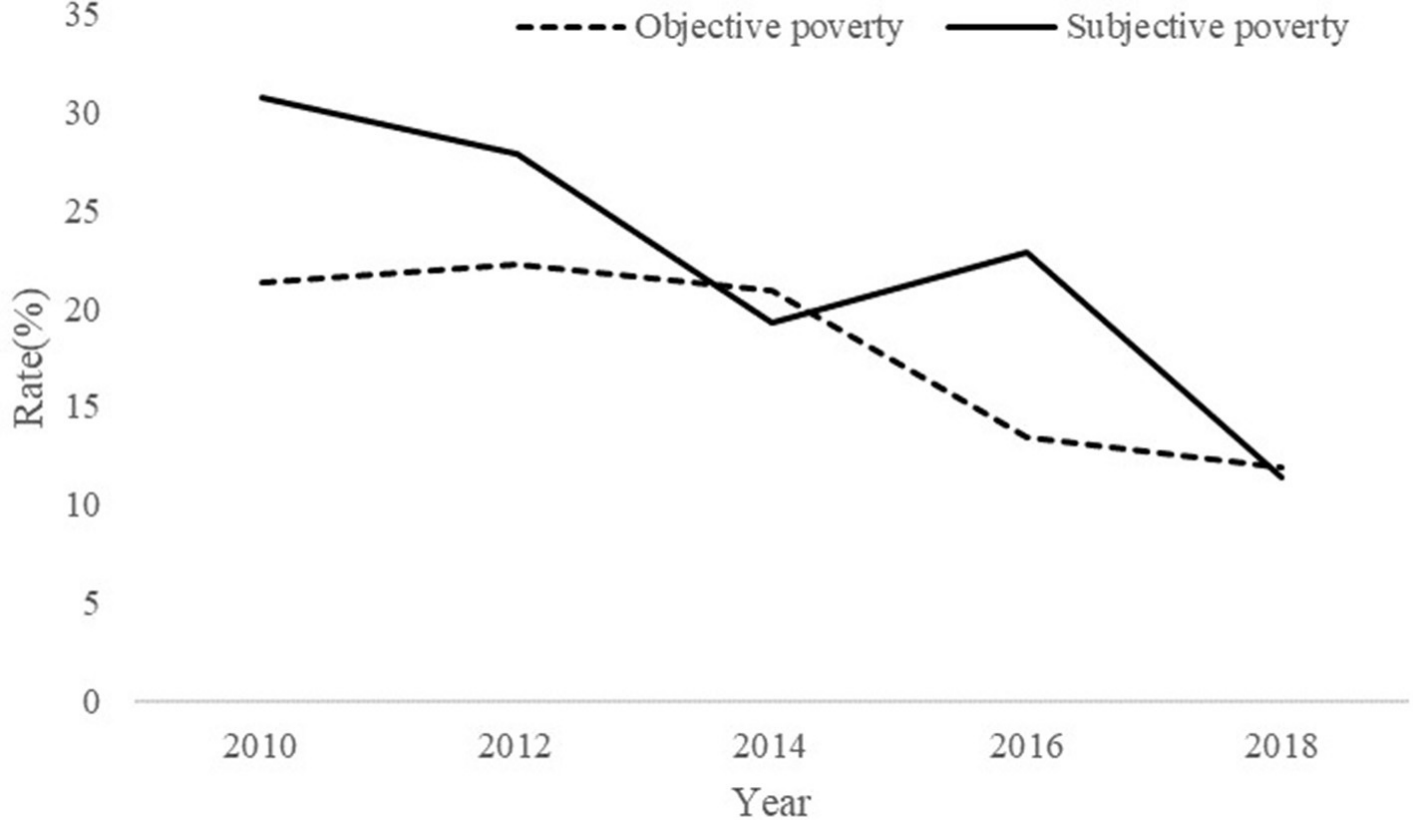
Rate of subjective poverty and objective poverty from 2010 to 2018.

Figure 1 shows the rate of subjective poverty and objective poverty from 2010 to 2018. The solid line represents the subjective poverty rate, while the dotted line is the rate of objective poverty every year. Both the rate of subjective poverty and objective poverty have decreased a lot in the past decade, with subjective poverty from 30.75% to 11.34% and objective poverty from 21.31% to 11.86%. However, the rate of subjective poverty is higher than that of objective poverty in most years, except for 2014 and 2018, while in these 2 years, the rate of subjective poverty is slightly lower than that of objective poverty.

### Duration of subjective poverty and physical health

Table 2 exhibits the OLS models 1–3 in the total sample examining the relationship between the duration of subjective poverty (2010–2016 wave) and SRH (2018 wave). The results in model 1 suggest that the duration of subjective poverty negatively predicts SRH (Coef. = −0.11, *p* < 0.01), net of working status, marriage, education BMI, gender, age, and rural. The results in model 2 show that after controlling present subjective poverty status in 2018, the duration of subjective poverty still has a significantly negative effect on SRH (Coef. = −0.09, *p* < 0.01). Model 3 furtherly controls the duration of objective poverty and present objective poverty status. The results in model 3 indicate that net of objective poverty, duration of subjective poverty still has a significantly negative association with individual's SRH (Coef. = −0.07, *p* < 0.01). The coefficients of duration objective poverty and present objective poverty are not statistically significant, indicating that subjective poverty is a stronger predictor than objective poverty.

**Table 2:** Duration of subjective poverty and SRH in the total sample.

	**Model 1**		**Model 2**		**Model 3**	
	**Coef**.	**S.E**.	**Coef**.	**S.E**.	**Coef**.	**S.E**.
Duration of subjective poverty (2010~2016)	−0.11***	0.01	−0.09***	0.01	−0.07***	0.01
Present subjective poverty (2018)			−0.35***	0.04	−0.34***	0.04
Duration of objective poverty (2010~2016)					−0.01	0.01
Present objective poverty (2018)					−0.07	0.04
Currently working	0.32***	0.03	0.29***	0.03	0.29***	0.03
**Education**						
Junior school and below	0.04	0.03	0.03	0.03	0.03	0.03
High school and technical secondary school	0.02	0.04	0.02	0.04	0.02	0.04
Junior college and above	−0.05	0.05	−0.02	0.05	0.04	0.06
**Marital status**						
Unmarried	−0.07	0.08	−0.07	0.08	−0.02	0.09
Other statuses	0.03	0.04	0.04	0.04	0.05	0.05
**BMI group**						
Underweight	−0.27***	0.05	−0.25***	0.05	−0.28***	0.06
Overweight	0.02	0.02	0.02	0.02	0.01	0.03
Obesity	−0.10***	0.04	−0.11***	0.04	−0.13***	0.04
Male	0.15***	0.02	0.15***	0.02	0.15***	0.03
**Region**						
Middle	0.10***	0.03	0.11***	0.03	0.14***	0.03
Eastern	0.12***	0.03	0.13***	0.03	0.15***	0.03
**Age group**						
36~45	−0.28***	0.05	−0.28***	0.05	−0.30***	0.05
46~55	−0.49***	0.05	−0.48***	0.05	−0.49***	0.05
56~65	−0.62***	0.05	−0.61***	0.05	−0.61***	0.05
66 and above	−0.72***	0.05	−0.72***	0.05	−0.71***	0.06
Rural	−0.07**	0.03	−0.05**	0.03	−0.04**	0.03
R2	0.0725		0.0765		0.0731	
Observations	11,963		11,808		9,604	

** and ***: significantly different from zero at the 0.05 and 0.01 level, respectively.

We furtherly computed the regression models in urban and rural samples separately. Table 3 exhibits the results in the urban sample. Model 1 indicates that the duration of subjective poverty negatively predicts personal SRH (Coef. = −0.06, *p* < 0.01). After controlling the present subjective poverty in model 2, the duration of subjective poverty still has a negative effect on SRH (Coef. = −0.03, *p* < 0.1). However, the results in model 3 suggest that the relationship between the duration of subjective poverty and SRH becomes insignificant after controlling the duration of objective poverty and present objective poverty status in the urban sample.

**Table 3:** Duration of subjective poverty and SRH in the urban sample.

	**Model 1**		**Model 2**		**Model 3**	
	**Coef**.	**S.E**.	**Coef**.	**S.E**.	**Coef**.	**S.E**.
Duration of subjective poverty (2010~2016)	−0.06***	0.02	−0.03*	0.02	−0.03	0.02
Present subjective poverty (2018)			−0.31***	0.07	−0.31***	0.07
Duration of objective poverty (2010~2016)					0.01	0.02
Present objective poverty (2018)					−0.14**	0.07
Currently working	0.31***	0.05	0.31***	0.05	0.31***	0.05
**Education**						
Junior school and below	0.14**	0.07	0.13*	0.07	0.16**	0.07
High school and technical secondary school	0.09	0.07	0.10	0.07	0.12	0.08
Junior college and above	0.11	0.08	0.10	0.08	0.14	0.09
**Marital status**						
Unmarried	−0.41***	0.13	−0.39***	0.13	−0.44***	0.14
Other statuses	0.09	0.06	0.11*	0.06	0.10	0.07
**BMI group**						
Underweight	−0.32***	0.10	−0.32***	0.10	−0.32***	0.10
Overweight	0.02	0.04	0.02	0.04	0.01	0.04
Obesity	−0.14**	0.06	−0.13**	0.06	−0.16**	0.06
Male	0.09**	0.04	0.09**	0.04	0.10**	0.04
**Region**						
Middle	−0.04	0.06	−0.03	0.06	−0.01	0.06
Eastern	−0.05	0.05	−0.06	0.05	−0.05	0.06
**Age group**						
36~45	−0.27***	0.08	−0.27***	0.08	−0.25***	0.09
46~55	−0.40***	0.08	−0.38***	0.08	−0.37***	0.09
56~65	−0.47***	0.08	−0.46***	0.08	−0.46***	0.09
66 and above	−0.59***	0.09	−0.58***	0.09	−0.57***	0.10
R2	0.0785		0.0834		0.0879	
Observations	3,525		3,496		3,024	

*, **, and ***: significantly different from zero at the 0.1, 0.05, and 0.01 level, respectively.

The results in Table 4 show the regression model in the rural sample. Model 1 indicates that the duration of subjective poverty negatively predicts SRH (Coef. = −0.14, *p* < 0.01) in the rural sample, net of working status, marriage, education BMI, gender, and age. The results in model 2 show that after controlling present subjective poverty status in 2018, the duration of subjective poverty still has a significantly negative effect on SRH (Coef. = −0.11, *p* < 0.01) for rural residents. The results in model 3 indicate that net of objective poverty, duration of subjective poverty has a significantly negative association with individual's SRH (Coef. = −0.10, *p* < 0.01). Consistent with the results in the total sample, the coefficients of duration objective poverty and present objective poverty are not statistically significant, indicating that subjective poverty is a stronger predictor than objective poverty in the rural sample.

**Table 4:** Duration of subjective poverty and SRH in the rural sample.

	**Model 1**		**Model 2**		**Model 3**	
	**Coef**.	**S.E**.	**Coef**.	**S.E**.	**Coef**.	**S.E**.
Duration of subjective poverty (2010~2016)	−0.14***	0.01	−0.11***	0.01	−0.10***	0.02
Present subjective poverty (2018)			−0.37***	0.05	−0.36***	0.05
Duration of objective poverty (2010~2016)					−0.04	0.02
Present objective poverty (2018)					−0.04	0.06
Currently working	0.35***	0.03	0.31***	0.04	0.30***	0.05
**Education**						
Junior school and below	0.01	0.03	−0.02	0.03	−0.03	0.04
High school and technical secondary school	0.01	0.05	0.001	0.05	−0.01	0.06
Junior college and above	−0.07	0.10	−0.85	0.10	−0.11	0.11
**Marital status**						
Unmarried	0.08	0.10	0.07	0.10	0.18	0.11
Other statuses	−0.003	0.06	−0.003	0.06	0.02	0.06
**BMI group**						
Underweight	−0.24***	0.06	−0.23***	0.06	−0.26***	0.07
Overweight	0.02	0.03	0.02	0.03	0.01	0.03
Obesity	−0.09*	0.05	−0.10**	0.05	−0.10*	0.05
Male	0.18***	0.03	0.18***	0.03	0.18***	0.03
**Region**						
Middle	0.13***	0.04	0.14***	0.04	0.16***	0.04
Eastern	0.18***	0.03	0.19***	0.03	0.21***	0.04
**Age group**						
36~45	−0.25***	0.06	−0.28***	0.06	−0.32***	0.07
46~55	−0.54***	0.05	−0.53***	0.05	−0.56***	0.06
56~65	−0.69***	0.06	−0.68***	0.06	−0.69***	0.07
66 and above	−0.76***	0.06	−0.76***	0.06	−0.75***	0.07
R2	0.0761		0.0800		0.0748	
Observations	8438		8312		6580	

*, **, and ***: significantly different from zero at the 0.1, 0.05, and 0.01 level, respectively.

Table 5 furtherly exhibits the relationship between experiencing different time points of subjective poverty and SRH in total, urban, and rural samples, respectively. The reference group is those who have not experienced subjective poverty in any of the survey waves. The results in the total sample indicate that as the time points increase, the negative effect of duration of subjective poverty on SRH also increases. Compared with those who have not experienced subjective poverty, the SRH score of people who experienced four time points is likely to decrease by 0.43. In the rural area, the effect could be even greater (Coef. = −0.54, *p* < 0.01). However, in the urban area, compared with those who did not experience subjective poverty from 2010 to 2016, those who experienced one, two, or three time points of subjective poverty have not been observed to have significantly worse health. Only those who experienced four time points of subjective poverty have significantly worse health (Coef. = −0.30, *p* < 0.01). In total, the duration of subjective poverty damages rural residents' physical health more than the urban residents, and the duration of subjective poverty is a stronger predictor of SRH than the duration of objective poverty.

**Table 5:** Different durations of subjective poverty and SRH.

	**Total sample**		**Urban sample**		**Rural sample**	
	**Coef**.	**S.E**.	**Coef**.	**S.E**.	**Coef**.	**S.E**.
Time points of subjective poverty (2010~2016)						
1 time point of subjective poverty	−0.08***	0.03	0.01	0.05	−0.12***	0.04
2 time points of subjective poverty	−0.14***	0.04	0.01	0.06	−0.21***	0.05
3 time points of subjective poverty	−0.13**	0.05	−0.01	0.08	−0.19***	0.07
4 time points of subjective poverty	−0.43***	0.08	−0.30***	0.10	−0.54***	0.11
Present subjective poverty (2018)	−0.34***	0.04	−0.30***	0.07	−0.35***	0.06
Duration of objective poverty (2010~2016)	−0.01	0.01	0.01	0.02	−0.04	0.02
Present objective poverty (2018)	−0.07	0.04	−0.13*	0.07	−0.04*	0.06
Currently working	0.29***	0.04	0.31***	0.05	0.30***	0.05
**Education**						
Junior school and below	0.03	0.03	0.15**	0.07	−0.02	0.04
High school and technical secondary school	0.02	0.04	0.12	0.08	−0.01	0.06
Junior college and above	−0.04	0.06	0.14	0.09	−0.11	0.11
**Marital status**						
Unmarried	−0.02	0.09	−0.43***	0.14	0.18	0.11
Other statuses	0.06	0.05	0.10	0.07	0.02	0.06
**BMI group**						
Underweight	−0.28***	0.06	−0.32***	0.11	−0.26***	0.07
Overweight	0.01	0.03	0.01	0.04	0.01	0.03
Obesity	−0.13***	0.04	−0.17***	0.06	−0.11*	0.05
Male	0.15***	0.03	0.10***	0.04	0.18***	0.03
**Region**						
Middle	0.14***	0.03	−0.01	0.06	0.16***	0.04
Eastern	0.15***	0.03	−0.05	0.06	0.21***	0.04
Age group						
36~45	−0.30***	0.05	−0.24***	0.09	−0.32***	0.07
46~55	−0.49***	0.05	−0.36***	0.09	−0.56***	0.06
56~65	−0.61***	0.05	−0.45***	0.09	−0.69***	0.07
66 and above	−0.71***	0.06	−0.57***	0.10	−0.75***	0.07
Rural	−0.04	0.03				
R2	0.0739		0.0901		0.0755	
Observations	9,604		3,024		6,580	

*, **, and ***: significantly different from zero at the 0.1, 0.05, and 0.01 level, respectively.

### Duration of subjective poverty and mental health

Table 6 exhibits the OLS models 1–3 in the total sample examining the relationship between the duration of subjective poverty (2010–2016 wave) and depressive symptoms (2018 wave). The results in model 1 suggest that the duration of subjective poverty positively predicts depressive symptoms (Coef. = 0.53, *p* < 0.01), net of working status, marriage, education BMI, gender, age, and rural. The results in model 2 show that after controlling present subjective poverty status in 2018, the duration of subjective poverty is still positively related to depressive symptoms (Coef. = 0.45, *p* < 0.01). Model 3 furtherly controls the duration of objective poverty and present objective poverty status. The results in model 3 indicate that net of objective poverty, duration of subjective poverty has a significantly positive association with individual's depressive symptoms (Coef. = 0.35, *p* < 0.01). One time point increase in duration of subjective poverty is likely related to a 0.35 increase in CES-D8 scores, while one time point increase in the duration objective poverty is related to a 0.18 increase in CES-D8 scores. It is apparent from our results that duration of subjective poverty has a greater effect on individual's mental health than the duration of objective poverty.

**Table 6:** Duration of subjective poverty and depressive symptoms in the total sample.

	**Model 1**		**Model 2**		**Model 3**	
	**Coef**.	**S.E**.	**Coef**.	**S.E**.	**Coef**.	**S.E**.
Duration of subjective poverty (2010~2016)	0.53***	0.04	0.45***	0.04	0.35***	0.04
Present subjective poverty (2018)			1.11***	0.15	1.05***	0.16
Duration of objective poverty (2010~2016)					0.18***	0.05
Present objective poverty (2018)					0.78***	0.15
Currently working	−0.10	0.11	−0.05	0.11	−0.17	0.12
**Education**						
Junior school and below	−0.69***	0.10	−0.66***	0.10	−0.54***	0.11
High school and technical secondary school	−0.94***	0.13	−0.92***	0.13	−0.72***	0.14
Junior college and above	−0.47***	0.17	−0.45***	0.17	−0.13	0.19
**Marital status**						
Unmarried	0.94***	0.30	0.93***	0.30	0.76**	0.33
Other statuses	1.69***	0.16	1.67***	0.16	1.59***	0.17
**BMI group**						
Underweight	0.83***	0.18	0.80***	0.18	0.83***	0.20
Overweight	−0.30***	0.08	−0.30***	0.08	−0.27***	0.09
Obesity	−0.29**	0.12	−0.27**	0.13	−0.26*	0.14
Male	−0.85***	0.08	−0.86***	0.08	−0.86***	0.08
**Region**						
Middle	−0.79***	0.10	−0.80***	0.10	−0.81**	0.11
Eastern	−1.02***	0.09	−1.02***	0.09	−0.96***	0.10
**Age group**						
36~45	0.36**	0.15	0.35**	0.15	0.38**	0.17
46~55	0.32**	0.14	0.26*	0.14	0.33*	0.17
56~65	0.37**	0.15	0.34**	0.15	0.44**	0.17
66 and above	−0.05	0.16	0.07	0.16	−0.05	0.19
Rural	0.81***	0.09	0.79***	0.09	0.73***	0.11
R2	0.1001		0.1049		0.1094
Observations	11,876		11,730		9,551

*, **, and ***: significantly different from zero at the 0.1, 0.05, and 0.01 level, respectively.

Table 7 shows the results of OLS regression of duration of subjective poverty and depressive symptoms in the urban sample. Different from SRH, the results in Table 7 indicate that the duration of subjective poverty plays a positive role in individual's depressive symptoms in the urban sample (Coef. = 0.15, *p* < 0.05), after controlling present subjective poverty, the duration of objective poverty, present objective poverty, and other covariates.

**Table 7:** Duration of subjective poverty and depressive symptoms in the urban sample.

	**Model 1**		**Model 2**		**Model 3**	
	**Coef**.	**S.E**.	**Coef**.	**S.E**.	**Coef**.	**S.E**.
Duration of subjective poverty (2010~2016)	0.30***	0.06	0.23***	0.07	0.15**	0.07
Present subjective poverty (2018)			0.88***	0.26	0.89***	0.27
Duration of objective poverty (2010~2016)					0.18***	0.07
Present objective poverty (2018)					0.92***	0.23
Currently working	−0.01	0.17	0.003	0.17	−0.16	0.18
**Education**						
Junior school and below	−0.87***	0.24	−0.84***	0.24	−0.56**	0.26
High school and technical secondary school	−1.27***	0.25	−1.25***	0.26	−0.89***	0.28
Junior college and above	−1.12***	0.27	−1.10***	0.28	−0.42	0.31
**Marital status**						
Unmarried	1.52**	0.63	1.47**	0.63	1.55**	0.66
Other statuses	1.57***	0.25	1.51***	0.25	1.41***	0.26
**BMI group**						
Underweight	1.08***	0.39	1.11***	0.39	1.14***	0.44
Overweight	−0.25*	0.13	−0.24*	0.13	−0.17	0.14
Obesity	−0.48**	0.21	−0.51**	0.21	−0.49**	0.22
Male	−0.76***	0.13	−0.77***	0.13	−0.88***	0.14
**Region**						
Middle	−0.51**	0.20	−0.54***	0.20	−0.62***	0.22
Eastern	−0.79***	0.19	−0.80***	0.19	−0.64***	0.21
**Age group**						
36~45	0.83***	0.27	0.28***	0.27	0.94***	0.30
46~55	0.54**	0.27	0.51*	0.27	0.85***	0.30
56~65	−0.001	0.29	−0.01	0.30	0.48	0.32
66 and above	−0.35	0.31	−0.35	0.32	0.13	0.35
R2	0.0769		0.0806		0.0933
Observations	3,507		3,480		3,011

*, **, and ***: significantly different from zero at the 0.1, 0.05, and 0.01 level, respectively.

The results in Table 8 show the regression models in the rural sample. Model 1 indicates that the duration of subjective poverty positively predicts depressive symptoms (Coef. = 0.61, *p* < 0.01) in the rural sample, net of working status, marriage, education BMI, gender, and age. The results in model 2 show that after controlling present subjective poverty status in 2018, the duration of subjective poverty still has a significantly negative effect on depressive symptoms (Coef. = 0.53, *p* < 0.01) for rural residents. The results in model 3 indicate that net of objective poverty, duration of subjective poverty has a significantly negative association with individual's depressive symptoms (Coef. = 0.46, *p* < 0.01). One unit increase in the duration of subjective poverty is related to 0.46 units increase in CES-D8 scores, while that of the duration of objective poverty is related to 0.20 units increase in CES-D8 scores.

**Table 8:** Duration of subjective poverty and depressive symptoms in the rural sample.

	**Model 1**		**Model 2**		**Model 3**	
	**Coef**.	**S.E**.	**Coef**.	**S.E**.	**Coef**.	**S.E**.
Duration of subjective poverty (2010~2016)	0.61***	0.05	0.53***	0.05	0.46***	0.05
Present subjective poverty (2018)			1.18***	0.18	1.11***	0.20
Duration of objective poverty (2010~2016)					0.20***	0.06
Present objective poverty (2018)					0.66***	0.19
Currently working	−0.38***	0.15	−0.32**	0.15	−0.32*	0.17
**Education**						
Junior school and below	−0.58***	0.11	−0.55***	0.11	−0.49***	0.12
High school and technical secondary school	−0.79***	0.17	−0.77***	0.17	−0.57***	0.19
Junior college and above	−0.32	0.31	−0.28	0.31	−0.08	0.37
**Marital status**						
Unmarried	0.71**	0.34	0.73**	0.34	0.43	0.37
Other statuses	1.75***	0.21	1.75***	0.21	1.72***	0.23
**BMI group**						
Underweight	0.71***	0.20	0.65***	0.20	0.71***	0.22
Overweight	−0.31***	0.10	−0.32***	0.10	−0.31***	0.11
Obesity	−0.23	0.15	−0.20	0.15	−0.18	0.17
Male	−0.89***	0.09	−0.90***	0.09	−0.86***	0.10
**Region**						
Middle	−0.91***	0.12	−0.90***	0.12	−0.83***	0.13
Eastern	−1.12***	0.11	−1.12***	0.11	−1.09***	0.12
**Age group**						
36~45	0.17	0.18	0.16	0.18	0.16	0.21
46~55	0.28*	0.17	0.21	0.17	0.17	0.20
56~65	0.53**	0.17	0.49***	0.17	0.47**	0.21
66 and above	0.17	0.19	−0.18	0.19	−0.15	0.23
R2	0.0974		0.1033		0.1040	
Observations	8,369		8,250		6,540	

*, **, and ***: significantly different from zero at the 0.1, 0.05, and 0.01 level, respectively.

Table 9 furtherly exhibits the relationship between experiencing different time points and subjective poverty and depressive symptoms in total, urban, and rural samples, respectively. The reference group is those who have not experienced subjective poverty in any of the survey waves. The results in the total sample indicate that as the time points increase, the negative effect of duration of subjective poverty on depressive symptoms also increases. Compared with those who have not experienced subjective poverty, the CES-D8 scores of people who experienced four time points are likely to increase by 1.26. In rural area, the effect could be even greater (Coef. = 1.47, *p* < 0.01). However, in the urban area, compared with those who did not experience subjective poverty from 2010 to 2016, those who experienced one, two, or three time points of subjective poverty have not been observed to have significantly higher CES-D8 scores. Only those who experienced four time points of subjective poverty have significantly higher CES-D8 scores (Coef. = 0.95, *p* < 0.05). In total, consistent with physical health, the duration of subjective poverty damages rural residents' mental health more than the urban residents.

**Table 9:** Different durations of subjective poverty and depressive symptoms.

	**Total sample**		**Urban sample**		**Rural sample**	
	**Coef**.	**S.E**.	**Coef**.	**S.E**.	**Coef**.	**S.E**.
**Time points of subjective poverty (2010** **~** **2016)**						
1 time point of subjective poverty	0.38***	0.10	0.26	0.18	0.44***	0.12
2 time points of subjective poverty	0.60***	0.13	0.14	0.21	0.81***	0.15
3 time points of subjective poverty	1.21***	0.18	0.29	0.29	1.68***	0.23
4 time points of subjective poverty	1.26***	0.29	0.95**	0.43	1.47***	0.39
Present subjective poverty (2018)	1.05***	0.16	0.87***	0.27	1.11***	0.20
Duration of objective poverty (2010~2016)	0.19***	0.05	0.18**	0.07	0.20***	0.06
Present objective poverty (2018)	0.78***	0.15	0.92***	0.23	0.66***	0.19
Currently working	−0.17	0.12	−0.16	0.18	−0.32*	0.17
**Education**						
Junior school and below	−0.54***	0.11	−0.56**	0.26	−0.49***	0.12
High school and technical secondary school	−0.73***	0.14	−0.87***	0.28	−0.58***	0.19
Junior college and above	−0.13	0.19	−0.42	0.31	−0.07	0.37
**Marital status**						
Unmarried	0.76**	0.33	1.52**	0.66	0.44	0.37
Other statuses	1.60***	0.17	1.42***	0.26	1.72***	0.23
**BMI group**						
Underweight	0.83***	0.20	1.12**	0.44	0.71***	0.22
Overweight	−0.27***	0.09	−0.17	0.14	−0.31***	0.11
Obesity	−0.26*	0.14	−0.48**	0.22	−0.18	0.17
Male	−0.86	0.08	−0.88***	0.14	−0.85***	0.10
**Region**						
Middle	−0.80***	0.11	−0.63***	0.22	−0.82***	0.13
Eastern	−0.96***	0.10	−0.64***	0.21	−1.09***	0.12
**Age group**						
36~45	0.38**	0.18	0.93***	0.30	0.15	0.21
46~55	0.32*	0.17	0.85***	0.30	0.16	0.20
56~65	0.44**	0.17	0.48	0.33	0.47**	0.21
66 and above	−0.06	0.19	−0.13	0.35	−0.16	0.23
Rural	0.97***	0.11				
R2	0.1097		0.0942		0.1047	
Observations	9551		3011		6540	

*, **, and ***: significantly different from zero at the 0.1, 0.05, and 0.01 level, respectively.

## Discussion

Most previous studies evaluated the effect of subjective poverty on health at one point of time, while from the life course perspective, the past duration of subjective poverty is as important as present subjective poverty to individual's health. Using longitudinal data to measure the duration of subjective poverty on individual's physical and mental health is of important significance. This current study fills in the research gap and provides evidence from China using a nationally representative database. We controlled present subjective poverty status, present objective poverty status, and duration of objective status to examine whether the duration of subjective poverty still had an impact on subsequent health. Both physical and mental health were measured in our analysis, and the heterogeneity between rural and urban areas was also analyzed. To the best of our knowledge, this is the first study to systematically measure the relationship between the duration of subjective poverty and individual's subsequent physical and mental health in China, net of present subjective poverty status and objective poverty status.

Our results contribute evidence that the duration of subjective poverty has a significantly negative effect on individual's physical health, especially for rural residents. The SRH scores of individuals who have experienced four time points of subjective poverty are likely to decrease by 0.30 in urban and 0.54 in rural, compared with those who have not experienced subjective poverty. The negative effect of the duration of subjective poverty is independent of that of objective poverty. Our results of subjective poverty on health are consistent with previous studies (13, 36). It has been largely documented that SSS or subjective poverty could be negatively related to personal physical health. Our finding confirms the existing ideas and provides more evidence from life course perspective. According to the life course perspective, chronically disadvantaged social status causes a quite different life course and capability to damage (37). On the one hand, a low social position could lead to a series of psychological stress (24). Being exposed to stress and lack of life resources for a long time make people who are in subjective poverty even more vulnerable when faced with diseases and other life events. A previous study published in the BMJ reported that stress-related disorders were robustly associated with a series of types of cardiovascular diseases, and the relationship was independent of family factors and disease history (38). On the other hand, being chronically subjective poverty could lead to unhealthy living habits, such as drinking because people usually choose to drink heavily as a way to cope with stress (39, 40). In addition, perceived stress has been documented to be significantly associated with other health-risk behaviors including low intake of fruit or vegetables, smoking, and physical inactivity (41). In this way, long standing of subjective poverty could induce long-term health-risk behaviors and indirectly engender bad physical health.

Our results also suggest that the duration of subjective poverty has a significantly negative effect on individual's mental health. The CES-D8 scores of individuals who have experienced four time points of subjective poverty are likely to increase by 0.95 in urban and 1.47 in rural, compared with those who have not experienced subjective poverty. The SSS is regarded to affect health through a psychological pathway (29). Self-perception of poverty or low income could bring multiple negative emotions such as stress, anxiety, and low self-esteem (42), while relatively higher social comparison engenders better self-esteem and fosters a sense of control, purpose, and meaningfulness in life (43). Emotion is strongly associated with mental health. Depression is related to reductions in positive emotion (44). Except for negative emotions, people who chronically live in subjective poverty have limited social support as well (45), while social support is an important contributor to mental health (46). In addition, another study also reported that people with low SSS usually had low level of health literacy and in case generated worse mental health (47). Beyond these studies focusing on cross-sectional subjective poverty or low SSS, our study provides evidence that long duration of subjective poverty can also negatively influence subsequent mental health, and the relationship is independent of present subjective poverty status, present objective poverty status, and duration of objective poverty status.

In addition, the findings of this study suggest that rural residents are more vulnerable to long duration of subjective poverty both in physical health and mental health than urban residents in China. In the rural area, residents who experienced one to four time points of subjective poverty appear to have an upgradient decrease in physical and mental health, while in the urban area, only those who experienced four time points of subjective poverty have significantly worse health, compared with those who did not experience subjective poverty from 2010 to 2016. It has been proposed earlier that rural poverty is more debilitating than urban poverty (48). From the aspect of the psychological pathway, in the rural area, poverty is regarded as more shameful than in the urban area (49). The poor living in the rural area has a greater feeling of isolation and failure. In this context, although long enduring subjective poverty brings damage to the health of both urban and rural residents, urban residents feel less stress, anxiety, and social isolation than the rural residents, which could help them to resist health restriction generated by subjective poverty. What is more, the living conditions in urban and rural areas are quite different in China. Although China's health system has developed a lot in the past decades, and the health service utilization and health supply have been greatly improved both in urban and rural areas, there is an increasing gap in health utilization between urban and rural residents (50). Rural residents are disadvantaged in all kinds of healthcare services, because of unbalanced health service supply (51). Health accessibility is vital for enhancing health equity. Therefore, we propose two possible explanations for the different vulnerability to the duration of subjective poverty for rural and urban residents. The first possible explanation is that in rural area, subjective poverty engenders more stress and shameful feeling than in the urban area; the second possible explanation is that urban residents have better access to health services which can help them defend against the health damages generated by subjective poverty.

Consistent with previous studies, this current research implies that subjective poverty is a stronger predictor of self-rated health than objective poverty. Our results indicate that after controlling for present subjective poverty and duration of subjective poverty, present objective poverty and duration of objective poverty have not observed significant effect on SRH in the rural area, and duration of objective poverty has not observed significant effect on SRH in the urban area. Plenty of studies have documented that subjective social status is more closely related to health outcomes than objective social status (13). Therefore, subjective social position and subjective poverty have gained more and more attention these years in certain western counties. The results of our study indicate that in most years, people with subjective poverty are much more than objective poverty. The past studies focusing only on objective poverty would neglect a large body of population who also lack multiple living resources. A study conducted in Pakistan found that measuring objective poverty only would miss some important information that subjective could include (52). For example, marital status is much more related to subjective poverty rather than objective poverty, and food insecurity is more likely to happen subjectively poor households according to the above research. Consequently, we propose subjective poverty should be attached more importance in the Chinese context. China has eliminated absolute poverty in 2020. The subjective poverty measurement would be a better and more powerful way to understand the poverty status of Chinese residents (25).

We want to caution against several limitations in this study. First, we only measured two health outcomes—SRH and depressive symptoms. Other health indicators were not included in our analysis. Although SRH and depressive symptoms are the two most acknowledged and widely used indicators to measure physical and mental health, our conclusion should be carefully generalized to other health outcomes. Second, our study could not provide a causal analysis so far. Future studies should try to provide causal analysis and explore the influencing mechanism between the duration of subjective poverty and health. Third, some potential confounding variables may not be included in our analysis, although we have included as many as variables according to existing literature.

## Conclusion

This study investigates whether the duration of subjective poverty predicts physical and mental health, after controlling present subjective poverty status, duration of objective poverty, present objective poverty status, and other covariates. The results show that a longer duration of subjective poverty negatively affects Chinese residents' physical and mental health, especially in rural area. Our study advocates researchers and policymakers pay more attention to the cumulative effect of subjective poverty on health in the Chinese context.

## Data availability statement

The datasets generated and analyzed during the current study were derived from the China Family Panel Study (CFPS). Researchers who want to use these data can visit: http://www.isss.pku.edu.cn/cfps/.

## Author contributions

DC and ZZ processed the data and were major contributors in writing the manuscript. GL and YR participated in the design of this study. CS and DZ acquired data and provided administrative support for data analysis. YZ, QD, and XZ were involved in revising the manuscript critically for important intellectual content. All authors have read and approved the final manuscript.

## Funding

This study was funded by the Project of Shaanxi Social Science Foundation (2017S024), the National Natural Science Foundation of China (71874137 and 72104195), and the China Postdoctoral Science Foundation (2021M702577).

## Conflict of interest

The authors declare that the research was conducted in the absence of any commercial or financial relationships that could be construed as a potential conflict of interest.

## Publisher's note

All claims expressed in this article are solely those of the authors and do not necessarily represent those of their affiliated organizations, or those of the publisher, the editors and the reviewers. Any product that may be evaluated in this article, or claim that may be made by its manufacturer, is not guaranteed or endorsed by the publisher.

## Footnotes

^1^ http://www.stats.gov.cn/
