# Supplementary material for: The relationship between duration of subjective poverty and health among Chinese adults: Evidence from the China Family Panel Study
Source: Front Public Health. 2022 Oct 5;10:939569. doi: 10.3389/fpubh.2022.939569 (PMC9581302; doi:10.3389/fpubh.2022.939569)
Supplement: Supplementary file 1 [file Data_Sheet_1.docx]

Table A1 VIF of regression models in Table 2, 3, 4, 6, 7 and 8

| Variables | Table 2 | | | Table 3 | | | Table 4 | | | Table 6 | | | Table 7 | | | Table 8 | | |
| --- | --- | --- | --- | --- | --- | --- | --- | --- | --- | --- | --- | --- | --- | --- | --- | --- | --- | --- |
|  | Model 1 | Model 2 | Model 3 | Model 1 | Model 2 | Model 3 | Model 1 | Model 2 | Model 3 | Model 1 | Model 2 | Model 3 | Model 1 | Model 2 | Model 3 | Model 1 | Model 2 | Model 3 |
| Duration of subjective poverty (2010-2016) | 1.07 | 1.14 | 1.20 | 1.14 | 1.24 | 1.30 | 1.07 | 1.14 | 1.17 | 1.07 | 1.14 | 1.20 | 1.14 | 1.24 | 1.29 | 1.07 | 1.14 | 1.17 |
| Present subjective poverty (2018) |  | 1.09 | 1.09 |  | 1.12 | 1.12 |  | 1.08 | 1.08 |  | 1.09 | 1.09 |  | 1.12 | 1.12 |  | 1.08 | 1.08 |
| Duration of objective poverty (2010-2016) |  |  | 1.37 |  |  | 1.64 |  |  | 1.20 |  |  | 1.37 |  |  | 1.64 |  |  | 1.20 |
| Present objective poverty (2018) |  |  | 1.20 |  |  | 1.36 |  |  | 1.15 |  |  | 1.20 |  |  | 1.36 |  |  | 1.14 |
| Currently working | 1.40 | 1.41 | 1.46 | 1.77 | 1.79 | 1.85 | 1.15 | 1.15 | 1.16 | 1.40 | 1.41 | 1.47 | 1.77 | 1.78 | 1.85 | 1.15 | 1.15 | 1.16 |
| Education |  |  |  |  |  |  |  |  |  |  |  |  |  |  |  |  |  |  |
| Junior school and below | 1.65 | 1.65 | 1.68 | 2.95 | 2.96 | 3.03 | 1.41 | 1.41 | 1.41 | 1.65 | 1.65 | 1.69 | 2.96 | 2.96 | 3.03 | 1.41 | 1.41 | 1.41 |
| High school and technical secondary school | 1.62 | 1.63 | 1.68 | 2.77 | 2.78 | 2.91 | 1.29 | 1.29 | 1.30 | 1.63 | 1.63 | 1.68 | 2.78 | 2.78 | 2.92 | 1.29 | 1.29 | 1.30 |
| Junior college and above | 1.63 | 1.63 | 1.72 | 2.80 | 2.82 | 3.07 | 1.12 | 1.12 | 1.13 | 1.63 | 1.63 | 1.72 | 2.81 | 2.82 | 3.08 | 1.12 | 1.12 | 1.13 |
| Marital status |  |  |  |  |  |  |  |  |  |  |  |  |  |  |  |  |  |  |
| Unmarried | 1.06 | 1.06 | 1.06 | 1.08 | 1.08 | 1.08 | 1.06 | 1.06 | 1.07 | 1.06 | 1.06 | 1.06 | 1.08 | 1.08 | 1.08 | 1.06 | 1.06 | 1.07 |
| Other status | 1.06 | 1.06 | 1.06 | 1.06 | 1.07 | 1.07 | 1.06 | 1.06 | 1.06 | 1.06 | 1.06 | 1.06 | 1.06 | 1.07 | 1.07 | 1.07 | 1.06 | 1.06 |
| BMI group |  |  |  |  |  |  |  |  |  |  |  |  |  |  |  |  |  |  |
| Underweight | 1.07 | 1.07 | 1.07 | 1.04 | 1.04 | 1.05 | 1.07 | 1.07 | 1.08 | 1.07 | 1.07 | 1.07 | 1.04 | 1.04 | 1.05 | 1.07 | 1.07 | 1.08 |
| Overweight | 1.12 | 1.12 | 1.12 | 1.12 | 1.12 | 1.13 | 1.12 | 1.12 | 1.12 | 1.12 | 1.12 | 1.12 | 1.12 | 1.12 | 1.13 | 1.12 | 1.12 | 1.11 |
| Obesity | 1.09 | 1.09 | 1.09 | 1.10 | 1.10 | 1.11 | 1.09 | 1.09 | 1.08 | 1.09 | 1.09 | 1.09 | 1.10 | 1.10 | 1.11 | 1.09 | 1.09 | 1.08 |
| Male | 1.11 | 1.10 | 1.11 | 1.09 | 1.09 | 1.10 | 1.13 | 1.12 | 1.12 | 1.11 | 1.10 | 1.11 | 1.09 | 1.09 | 1.09 | 1.12 | 1.12 | 1.12 |
| Region |  |  |  |  |  |  |  |  |  |  |  |  |  |  |  |  |  |  |
| Middle | 1.63 | 1.63 | 1.64 | 2.30 | 2.29 | 2.41 | 1.48 | 1.48 | 1.47 | 1.63 | 1.63 | 1.64 | 2.29 | 2.29 | 2.40 | 1.48 | 1.48 | 1.47 |
| Eastern | 1.67 | 1.68 | 1.71 | 2.30 | 2.29 | 2.44 | 1.52 | 1.52 | 1.53 | 1.67 | 1.67 | 1.71 | 2.29 | 2.29 | 2.44 | 1.52 | 1.52 | 1.52 |
| Age group |  |  |  |  |  |  |  |  |  |  |  |  |  |  |  |  |  |  |
| 36~45 | 2.92 | 2.91 | 3.14 | 3.30 | 3.30 | 3.56 | 2.80 | 2.80 | 3.00 | 2.92 | 2.92 | 3.15 | 3.31 | 3.31 | 3.58 | 2.80 | 2.80 | 3.01 |
| 46~55 | 4.11 | 4.10 | 4.54 | 4.28 | 4.27 | 4.70 | 4.09 | 4.08 | 4.51 | 4.12 | 4.11 | 4.55 | 4.30 | 4.29 | 4.74 | 4.09 | 4.08 | 4.51 |
| 56~65 | 3.86 | 3.84 | 4.30 | 4.73 | 4.72 | 5.35 | 3.67 | 3.65 | 4.04 | 3.87 | 3.85 | 4.31 | 4.75 | 4.75 | 5.39 | 3.67 | 3.65 | 4.04 |
| 66 and above | 4.11 | 4.08 | 4.52 | 5.67 | 5.69 | 6.31 | 3.69 | 3.64 | 4.03 | 4.12 | 4.09 | 4.53 | 5.69 | 5.71 | 6.34 | 3.69 | 3.65 | 4.04 |
| Rural | 1.43 | 1.44 | 1.60 |  |  |  |  |  |  | 1.43 | 1.44 | 1.61 |  |  |  |  |  |  |

Table A2 VIF of regression models in Table 5 and 9

| Variables | Table 5 | | | Table 9 | | |
| --- | --- | --- | --- | --- | --- | --- |
|  | Total sample | Urban sample | Rural sample | Total sample | Urban sample | Rural sample |
| Time points of subjective poverty (2010-2016) |  |  |  |  |  |  |
| 1 time point of subjective poverty | 1.18 | 1.21 | 1.17 | 1.18 | 1.21 | 1.17 |
| 2 time points of subjective poverty | 1.20 | 1.25 | 1.19 | 1.20 | 1.25 | 1.19 |
| 3 time points of subjective poverty | 1.15 | 1.18 | 1.14 | 1.15 | 1.18 | 1.14 |
| 4 time points of subjective poverty | 1.11 | 1.18 | 1.08 | 1.11 | 1.18 | 1.08 |
| Present subjective poverty (2018) | 1.09 | 1.12 | 1.09 | 1.10 | 1.12 | 1.09 |
| Duration of objective poverty (2010-2016) | 1.37 | 1.65 | 1.21 | 1.37 | 1.66 | 1.20 |
| Present objective poverty (2018) | 1.20 | 1.36 | 1.15 | 1.20 | 1.36 | 1.15 |
| Currently working | 1.46 | 1.85 | 1.16 | 1.47 | 1.85 | 1.16 |
| Education |  |  |  |  |  |  |
| Junior school and below | 1.69 | 3.03 | 1.41 | 1.69 | 3.03 | 1.41 |
| High school and technical secondary school | 1.69 | 2.92 | 1.30 | 1.69 | 2.93 | 1.30 |
| Junior college and above | 1.72 | 3.08 | 1.13 | 1.72 | 3.09 | 1.13 |
| Marital status |  |  |  |  |  |  |
| Unmarried | 1.06 | 1.08 | 1.07 | 1.06 | 1.08 | 1.07 |
| Other status | 1.06 | 1.08 | 1.06 | 1.07 | 1.07 | 1.06 |
| BMI group |  |  |  |  |  |  |
| Underweight | 1.07 | 1.05 | 1.08 | 1.07 | 1.05 | 1.08 |
| Overweight | 1.12 | 1.13 | 1.12 | 1.12 | 1.13 | 1.11 |
| Obesity | 1.09 | 1.11 | 1.08 | 1.09 | 1.11 | 1.08 |
| Male | 1.11 | 1.10 | 1.13 | 1.11 | 1.10 | 1.13 |
| Region |  |  |  |  |  |  |
| Middle | 1.64 | 2.41 | 1.47 | 1.64 | 2.41 | 1.47 |
| Eastern | 1.71 | 2.44 | 1.53 | 1.71 | 2.44 | 1.53 |
| Age group |  |  |  |  |  |  |
| 36~45 | 3.15 | 3.57 | 3.00 | 3.16 | 3.59 | 3.01 |
| 46~55 | 4.54 | 4.72 | 4.51 | 4.56 | 4.76 | 4.51 |
| 56~65 | 4.30 | 5.35 | 4.04 | 4.31 | 5.39 | 4.04 |
| 66 and above | 4.53 | 6.32 | 4.04 | 4.54 | 6.36 | 4.04 |
| Rural | 1.60 |  |  | 1.61 |  |  |
